# Myocardial Stiffness Evaluation Using Noninvasive Shear Wave Imaging in Healthy and Hypertrophic Cardiomyopathic Adults

**DOI:** 10.1016/j.jcmg.2018.02.002

**Published:** 2019-07

**Authors:** Olivier Villemain, Mafalda Correia, Elie Mousseaux, Jérome Baranger, Samuel Zarka, Ilya Podetti, Gilles Soulat, Thibaud Damy, Albert Hagège, Mickael Tanter, Mathieu Pernot, Emmanuel Messas

**Affiliations:** a Institut Langevin, ESPCI, CNRS, Inserm U979, PSL Research University, Paris, France; b Hôpital Européen Georges Pompidou, Université Paris Descartes, Cardio-Vascular Departement, UMR 970, Paris, France; c Hôpital Européen Georges Pompidou, Université Paris Descartes, Département de Radiologie, INSERM U970, Paris, France; d Department of Cardiology, AP-HP, Henri Mondor Teaching Hospital, Créteil, France

**Keywords:** diastolic function, echocardiography, myocardial stiffness, myocardium, DT, deceleration time, ECV, extracellular volume, FA, fractional anisotropy, HCM, hypertrophic cardiomyopathy, HF, heart failure, HFpEF, heart failure with preserved ejection function, HV, healthy volunteer, IVRT, isovolumic relaxation time, LGE, late gadolinium enhancement, MS, myocardial stiffness, SWI, shear wave imaging, Vp, transmitral flow propagation velocity

## Abstract

**Objectives:**

The goal of our study was to investigate the potential of myocardial shear wave imaging (SWI) to quantify the diastolic myocardial stiffness (MS) (kPa) noninvasively in adult healthy volunteers (HVs) and its physiological variation with age, and in hypertrophic cardiomyopathy (HCM) populations with heart failure and preserved ejection function (HFpEF).

**Background:**

MS is an important prognostic and diagnostic parameter of the diastolic function. MS is affected by physiological changes but also by pathological alterations of extracellular and cellular tissues. However, the clinical assessment of MS and the diastolic function remains challenging. SWI is a novel ultrasound-based technique that has the potential to provide intrinsic MS noninvasively.

**Methods:**

We prospectively included 80 adults: 60 HV (divided into 3 groups: 20- to 39-year old patients [n = 20]; 40- to 59-year-old patients [n = 20]; and 60- to 79-year-old patients [n = 20]) and 20 HCM-HFpEF patients. Echocardiography, cardiac magnetic resonance imaging and biological explorations were achieved. MS evaluation was performed using an ultrafast ultrasound scanner with cardiac phased array. The fractional anisotropy of MS was also estimated.

**Results:**

MS increased significantly with age in the HV group (the mean MS was 2.59 ± 0.58 kPa, 4.70 ± 0.88 kPa, and 6.08 ± 1.06 kPa for the 20- to 40-year-old, 40- to 60-year-old, and 60- to 80-year-old patient groups, respectively; p < 0.01 between each group). MS was significantly higher in HCM-HFpEF patients than in HV patients (mean MS = 12.68 ± 2.91 kPa vs. 4.47 ± 1.68 kPa, respectively; p < 0.01), with a cut-off at 8 kPa (area under the curve = 0.993; sensitivity = 95%, specificity = 100%). The fractional anisotropy was lower in HCM-HFpEF (mean = 0.133 ± 0.073) than in HV (0.238 ± 0.068) (p < 0.01). Positive correlations were found between MS and diastolic parameters in echocardiography (early diastolic peak/early diastolic mitral annular velocity, r = 0.783; early diastolic peak/transmitral flow propagation velocity, r = 0.616; left atrial volume index, r = 0.623) and with fibrosis markers in cardiac magnetic resonance (late gadolinium enhancement, r = 0.804; myocardial T1 pre-contrast, r = 0.711).

**Conclusions:**

MS was found to increase with age in healthy adults and was significantly higher in HCM-HFpEF patients. Myocardial SWI has the potential to become a clinical tool for the diagnostic of diastolic dysfunction. (Non-invasive Evaluation of Myocardial Stiffness by Elastography [Elasto-Cardio]; NCT02537041)

## Graphical abstract

**Figure:**
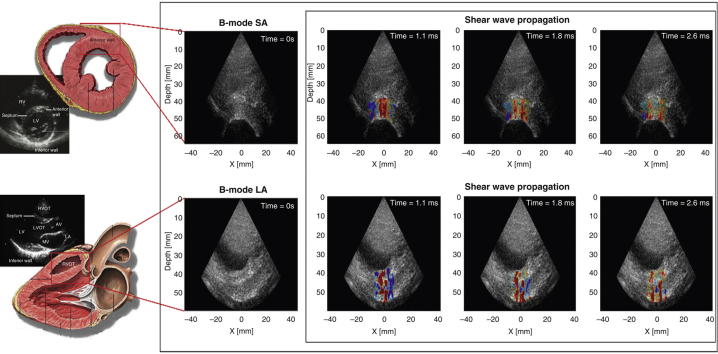

Myocardial stiffness (MS) is known to play a key role in diastolic left ventricular (LV) function (1). Abnormalities in LV relaxation and MS are one of the key pathophysiological mechanisms (2) in heart failure patients with preserved ejection fraction (HFpEF). Hypertrophic cardiomyopathy (HCM) is also associated to severe diastolic dysfunction mainly due to fibrosis and fiber disarray (3). Moreover, MS is also affected by aging due to progressive physiological changes and cellular and extracellular matrix alterations. However, as the clinical assessment of MS and of the diastolic function is still challenging (4), the study of MS remained limited to invasive explorations (5).

In a general view, the assessments of diastolic function can be divided into those that reflect the process of active/auxotonic relaxation (depending on filling load and afterload) and those that reflect passive stiffness (independent of load conditions) (6). In clinical practice, biological parameters are correlated with ventricular filling pressures (e.g., brain natriuretic protein [BNP]) (7), echocardiographic parameters are identified to assess the auxotonic relaxation and/or the filling pressure, and cardiac magnetic resonance (CMR) imaging offers tools to evaluate myocardial fibrosis (late enhancement gadolinium [LGE]) (8), or the collagen volume fraction (pre-post contrast T1 mapping or extracellular volume fraction [ECV]) 9, 10. However, noninvasive estimation of passive stiffness remains challenging. To date, cardiac catheterization is the only validated option to assess the passive stiffness clinically, through the compliance estimation thanks to the pressure-volume loops (11). But the risks for the patients, the necessary equipment, and the costs of these interventions make this examination unfeasible in daily clinical practice.

Shear wave imaging (SWI) is an ultrasound-based technique for quantitative, local, and noninvasive mapping of soft tissue's stiffness. The clinical impact of SWI has been shown during the last decade in the field of breast lesions (12) and liver (13) imaging. Quantification of MS using SWI has also been investigated extensively on animal models in previous studies (14). SWI was compared to invasive gold standard parameters (15) derived from pressure-volume loops and was shown to quantify the end-diastolic MS (i.e., passive stiffness) accurately. More recently, the clinical feasibility and reproducibility of transthoracic SWI was shown on a small group of healthy volunteers (HVs) (16) and on pediatric patients (17). The next step is to show the clinical interest and contribution of this technology for the assessment of diastolic MS in adults and its impact on diastolic LV function. Unlike other imaging techniques, echocardiography is inexpensive, widely available, and can be performed in real-time at the patient bedside allowing monitoring of the heart structure and function.

In this study, we aimed to perform the first clinical proof of concept of noninvasive MS evaluation on normal and pathological patients. More specifically, the goals of our study were: 1) to quantify MS noninvasively with SWI in a healthy adult population to establish values of MS and its dependence with age; 2) to compare it to severely altered MS in HCM patients with HFpEF; and 3) to investigate the correlation of MS with conventional echocardiography and CMR index of diastolic function.

## Methods

### Study patients and design

This was a prospective study conducted at the Hôpital Européen Georges Pompidou, Paris, France. A population of HVs was contacted and recruited by the Clinical Investigation Center. HV-specific inclusion criteria were: no history of heart failure symptoms, LV ejection fraction (EF) >50%, early diastolic peak (E)/early diastolic mitral annular velocity (e′) <13, as well as normal values of BNP (<35 pg/ml). Three age groups were composed within the recruited HVs: 20- to 39-year-old patients, 40- to 59-year-old patients, and 60- to 79-year-old patients. Exclusion criteria included systolic blood pressure (SBP) ≥140 mm Hg or/and diastolic blood pressure (DBP) ≥90 mm Hg, any persistent cardiac arrhythmia, more than moderate valvular disease, any relevant coronary artery diseases, any contraindication to CMR, and an echogenicity.

Patients with clinical, genetic, and echocardiographic evidence for sarcomeric HCM with HFpEF (HCM-HFpEF group) were included. HCM-HFpEF patients were identified according to the consensus of the European Society of Cardiology 18, 19, using specific inclusion criteria: wall thickness ≥15 mm in 1 or more LV myocardial segments; sarcomeric protein gene mutation identified; LVEF >50%; New York Heart Association (NYHA) functional class ≥II; at least 1 hospitalization for acute heart failure; and E/e′ ≥13 or E/e′ 8 to 13 combined with elevated BNP (>35 pg/ml).

All subjects included in the study underwent clinical explorations, biological explorations (hematocrit, C-reactive protein, BNP), an echocardiography, a CMR and a cardiac SWI. All explorations were performed on the same day. Three months later and if no clinical event was noted (symptoms of heart failure, hospitalization for cardiac cause, modification of weight or blood pressure), a second echocardiography, and cardiac SWI were realized to estimate the reproducibility on 5 patients per HV subgroup, randomly selected (n = 15).

The study was approved by the local ethics committee, and all patients gave written informed consent (Non-Invasive Evaluation of Myocardial Stiffness by Elastography [Elasto-Cardio]; NCT02537041).

### Imaging procedures

#### Echocardiography

Echocardiographic explorations were performed on a Vivid 9 system (General Electric Healthcare, Chalfont St. Giles, Great Britain). Mitral valve inflow pattern (E and A velocity), E-wave deceleration time (E-wave DT), isovolumic relaxation time (IVRT), transmitral flow propagation velocity (Vp), septal mitral valve annular velocities (e′ and a′), as well as pulmonary veins S-wave on D-wave ratio (PV S/D ratio) were recorded in an apical 4-chamber view, to assess the markers of diastolic function according to American Society of Echocardiography guidelines (20). Global and septal longitudinal strain was also performed by the Speckle Tracking 2D Strain software of General Electrics, directly on the Vivid 9 system. Data were analyzed from stored images by experienced operators (O.V. and A.H.) who were unaware of other test results. Measurements were made over 3 cardiac cycles; the average was used for statistical analysis.

#### CMR

The CMR protocol consisted of cine-sequences, T1-weighted spin-echo, and 2-dimensional inversion recovery gradient echo sequences for late enhancement assessment after gadobutrol administration (LGE). Post contrast T1 times (T1 mapping) was performed with a modified Look-Locker inversion recovery sequence with a 3(3)5 scheme before and 15 min after contrast application (21). Mapping was performed over all available short-axis slices. Extracellular volume (ECV) fraction was calculated on the basis of the combination of pre- and post-contrast T1 mapping data according to the approach proposed by Rommel et al. (10). All acquisitions were consistent with the Society for Cardiovascular Magnetic Resonance published guidelines (22). Data were interpreted by 2 experienced readers (E.M. and G.S.) who were unaware of the subjects’ clinical information and the results of other diagnostic tests.

#### MS measured by SWI

SWI is based on the remote generation of shear waves in soft tissue by acoustic radiation force combined with ultrasonic ultrafast imaging of the shear wave propagation (5,000 images/s), using the same ultrasonic transducer (Figure 1, Video 1) (23). This modality has already been described in previous works 14, 15, and is also described in more details in the Supplemental Appendix. In this study, a phased array probe (2.75-MHz central frequency, SuperSonic Imagine, Aix-en-Provence, France) connected to an ultrafast ultrasound scanner (Aixplorer, SuperSonic Imagine) was used. A conventional real-time echocardiographic image was used to position the probe. The focus of the acoustic radiation force generation was adjusted manually by the operator on the myocardial wall. The operator than launched the SWI acquisition that lasted approximately 1 s.Figure 1Myocardial Shear Wave ImagingB-mode and shear wave elastography imaging examples of a healthy volunteer (HV). Shear wave propagation in short- and long-axis views of a HV (tissue axial velocity images). Also see Video 1. AV = atrioventricular; LA = left atrium; LV = left ventricle; LVOT = left ventricular outflow tract; MV = mitral valve; RV = right ventricle; RVOT = right ventricular outflow tract; SA = short axis view.
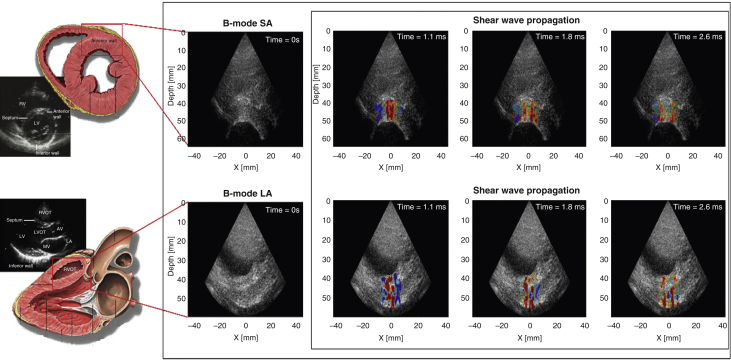

**Figure:**
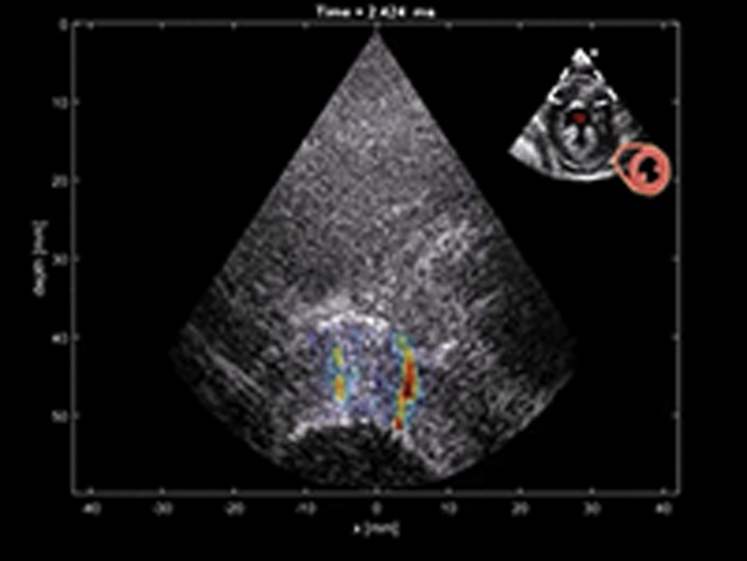
Video 1

The explored myocardial segment was the anteroseptal basal segment (ASB segment). It was evaluated in 2 orthogonal axes (short- and long-axis views) (Figure 1). Short-axis measurements were used to derive the shear modulus, whereas long-axis and short-axis values were used to compute the fractional anisotropy. All acquisitions were performed at end-diastole and triggered by an electrocardiogram. The 30 frames recorded after the push were post-processed to visualize the shear wave and compute the speed.

Data were interpreted off-line by 1 experienced reader (O.V.) who was unaware of the subjects’ clinical information and the results of other diagnostic tests.

#### Fractional anisotropy

Similar to any fiber-composed muscular tissue, the myocardium presents a significant anisotropy of its elastic properties. Consequently, MS is expected to be higher when measured along the fibers, which are mainly oriented along the circumferential direction in the mid-wall layer. To evaluate the degree of anisotropy in the myocardium, the fractional anisotropy (FA) was computed. FA was defined using two shear wave speed measurements performed in orthogonal propagation directions (long-axis and short-axis views) using the formula published by Lee et al. (24). More details on the method are given in the Supplemental Appendix.

### Statistical analysis

Data for continuous variables are presented as mean ± SD, if normally distributed, or as median and interquartile range if non-normally distributed. Categorical variables are presented as frequencies and percentages. Comparisons between groups were made using chi-square tests for categorical variables. Continuous variables were compared with unpaired Student’s *t* tests or the nonparametric Mann-Whitney U test where appropriate. Univariate and stepwise multivariate linear regression analyses were performed to identify predictors of r (standardized coefficient of linear regression). Receiver-operating characteristic (ROC) curves and area under the curve (AUC) were computed to assess the effectiveness of MS to predict healthy or pathologic subjects. Reproducibility of MS estimation (3 months after the first estimation) was tested by the Bland-Altman limits of agreement. The reproducibility coefficient was calculated as 1.96 × the SD of the differences, as proposed by Bland and Altman (25). All the analyses were conducted using Medcalc (MedCalc Software, Mariakerke, Belgium).

## Results

### Population characteristics

**Figure 2:**
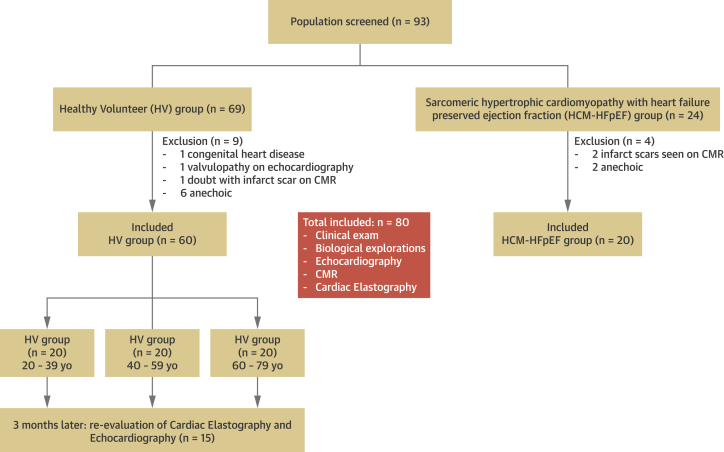
Study Flowchart The study was performed on 60 healthy volunteer and 20 HCM patients. CMR = cardiac magnetic resonance; HCM = hypertrophic cardiomyopathy; HFpEF = heart failure with preserved ejection fraction.

A total of 93 subjects (69 HV and 24 HCM-HFpEF) were prospectively screened for inclusion into the study (Figure 2). Eight patients from the HV group were excluded based on the exclusion criteria (1 congenital heart disease and 1 valvulopathy on echocardiography, 1 doubt on infract scar on CMR, 6 anechoic). Two patients of the HCM-HFpEF group were excluded based on the exclusion criteria (2 infarct scars seen on CMR, 2 anechoic). Finally, 80 subjects were included: HCM-HFpEF group (n = 20), HV 20-year-old to 39-year-old group (n = 20), HV 40- to 59-year-old group (n = 20), and HV 60- to 79-year-old group (n = 20) (Figure 2).

The molecular genetic causes for the HCM-HFpEF group were: 8 mutations of MYH7, 6 mutations of MYBPC3, 2 mutations of TNNT2, 1 mutation of TPM1, 1 mutation of TNNI3, 1 mutation of MYL3, and 1 mutation of MYL2.

The subjects’ baseline characteristics including clinical characteristics, laboratory data, echocardiographic results, and CMR results are shown in Table 1.

**Table 1:** Population Characteristics

	Healthy Volunteer (n = 60)	HCM-HFpEF (n = 20)	p Value
Patient			
Age, yrs	50.6 ± 16.9	57 ± 17.5	0.22
Sex, M/F	31/29	17/3	<0.01
BMI, kg/m^2^	23.6 ± 2.9	24.8 ± 3.6	0.74
Systolic BP, mm Hg	117 ± 10	115 ± 11	0.41
Diastolic BP, mm Hg	70 ± 7	75 ± 6	0.47
NYHA functional class I	52	0	<0.01
NYHA functional class II	8	14	<0.01
NYHA functional class III to IV	0	6	<0.01
Biology (blood explorations)			
CRP, mg/l	1.4 ± 1.1	1.9 ± 0.8	0.55
NTproBNP, pg/ml	16 ± 9	365 ± 144	<0.01
Hematocrit, %	43 ± 2	40 ± 2	0.03
Echocardiography parameters			
LA surface, 4 cavities view, cm^2^	16.1 ± 4	29.3 ± 7.1	<0.01
LAVI, ml/m^2^	25.9 ± 8.7	43.3 ± 18.6	<0.01
LAVI > 34 ml/m^2^, %	12/60 (20)	15/20 (75)	<0.01
LVEF, %	68 ± 9.9	66 ± 7.9	0.67
LVEDD, mm	45.9 ± 4.8	49.9 ± 5.7	0.47
LVESD, mm	28.3 ± 4.8	24.6 ± 3.2	0.22
LV mass/BSA, g/m^2^	70.6 ± 20	125 ± 34	<0.01
LV GS, %	17.4 ± 2.4	14.6 ± 3.1	<0.01
ASB segment GS, %	16.9 ± 2.2	6.4 ± 3.6	<0.01
ASB segment end diastolic thickness, mm	5.9 ± 1.4	20.8 ± 5.1	<0.01
Peak E-wave, cm/s	74.8 ± 17.6	88.3 ± 30.2	<0.01
E/A	1.4 ± 0.5	1.1 ± 0.4	<0.01
e’ septal, cm/s	13.8 ± 4.1	5.8 ± 1.9	<0.01
E/e’	5.9 ± 2.4	16.1 ± 6.5	<0.01
e’/a’ septal	1.6 ± 0.8	1.3 ± 0.9	0.29
E-wave DT, ms	179 ± 60	238 ± 62	<0.01
IVRT, ms	94 ± 17	144 ± 31	<0.01
Vp, cm/s	50.4 ± 7.4	29.2 ± 5.5	<0.01
E/Vp	1.3 ± 0.3	3.4 ± 1.5	<0.01
PV S/D ratio	1.2 ± 0.3	0.7 ± 0.3	<0.01
Cardiac magnetic resonance			
ASB segment end diastolic thickness, mm	5.7 ± 1.4	18.3 ± 3.4	<0.01
LV mass/LVED volume, g/ml	0.75 ± 0.17	2.1 ± 0.51	<0.01
Myocardial T1 pre-contrast, ms	1,217 ± 49	1,299 ± 80	<0.01
Blood T1 pre-contrast, ms	1,738 ± 102	1,694 ± 67	0.17
Myocardial T1 post-contrast, ms	440 ± 52	395 ± 60	0.02
Blood T1 post-contrast, ms	247 ± 41	230 ± 50	0.08
Focal LGE present (ASB segment), %	0/60 (0)	16/20 (80)	<0.01
Extracellular volume fraction, %	24.5 ± 3.7	27.2 ± 4.1	<0.01

Values are mean ± SD or n/N (%).

A = late diastolic peak (pulsed-wave Doppler); a’ = late diastolic mitral annular velocity by Doppler tissue imaging; ASB = anteroseptal basal; BMI = body mass index; BP = blood pressure; BSA = body surface area; CRP = C-reactive protein; DT = deceleration time; E = early diastolic peak (pulsed-wave Doppler); e’ = early diastolic mitral annular velocity by Doppler tissue imaging; GS = global strain; HCM = hypertrophic cardiomyopathy; HFpEF = heart failure with preserved ejection fraction; IVRT = isovolumic relaxation time; LA = left atrium; LAVI = left atrium volume index; LGE = late gadolinium enhancement; LVEF = left ventricle ejection fraction; LVEDD = left ventricle end-diastolic diameter; LVESD = left ventricle end-systolic diameter; NYHA = New York Heart Association; NTproBNP = N-terminal pro brain natriuretic peptide; PV S/D ratio = pulmonary veins velocities; Vp = transmitral flow propagation velocity.

There is no statistical difference between the HV group and the HCM-HFpEF group in terms of age (p = 0.22), body mass index (BMI) (p = 0.74), and BP (SBP, p = 0.41; DBP, p = 0.47). In both groups, there was no diabetic patient.

Concerning the HCM-HFpEF group, 6 patients (30%) had a New York Heart Association (NYHA) functional class ≥III (4 patients were class III, 2 patients were class IV). Twenty patients (100%) had a BNP >35 pg/ml. Regarding echocardiographic results, LV mass index was significantly higher than the HV group (125 ± 34 g/m^2^ vs. 70.6 ± 20 g/m^2^; p < 0.01), the ASB segment was significantly thicker than the HV group (20.8 ± 5.1 mm vs. 5.9 ± 1.4 mm; p < 0.01), with a segment strain lower than the HV group (−6.4 ± 3.6% vs. −16.9 ± 2.2%; p < 0.01). All the main diastolic function parameters were significantly different (p < 0.01) than those of the HV group (E/A; e′, E/e′, E-wave DT, IVRT, Vp, E/Vp, PV S/D ratio). Regarding CMR results, 16 of 20 (80%) had an LGE on the ASB segment (p < 0.01). There was a difference between HV and HCM-HFpEF groups concerning myocardial T1 post-contrast (p = 0.02) and ECV (p < 0.01).

### Myocardial stiffness results

#### HV group

**Figure 3:**
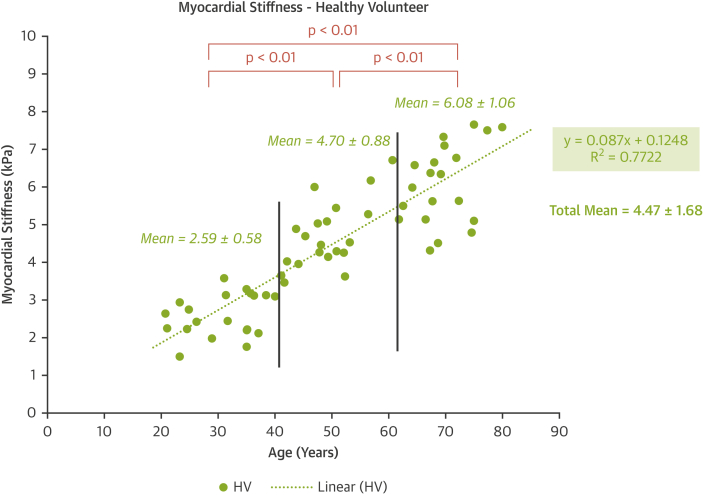
Myocardial Stiffness for Healthy Volunteers Myocardial stiffness measured in HV as a function of age. Abbreviation as in Figure 1.

The mean MS for the HV group was 4.47 ± 1.68 kPa (Figure 3). No patient from the HV group had an MS larger than 8 kPa. The mean MS was 2.59 ± 0.58 kPa for the 20- to 39-year-old HV group, 4.70 ± 0.88 kPa for the 40- to 59-year-old HV group, and 6.08 ± 1.06 kPa for the 60- to 79-year-old HV group. There was a statistical significant difference between all age groups (p < 0.01).

#### Myocardial stiffness dependence on age

A strong increase in MS with age was found (Figure 3). The correlation between age (x) and MS (y) values was robust (y = 0.087x + 0.1248; r^2^ = 0.77; p < 0.01). A multivariate linear regression analysis (including sex, age, BMI, hazard ratio SBP, and DBP) showed that age was the only clinical parameter correlated with MS (age, p < 0.01; sex, p = 0.77; BMI, p = 0.98; HR, p = 0.88; SBP, p = 0.33; DBP, p = 0.63). The correlation of echocardiographic parameters and age was lower: E/A, r^2^ = 0.30; E/e′, r^2^ = 0.23; E/Vp r^2^ = 0.01 (Supplemental Figure 1). In univariate analysis for the HV group, there was no correlation between LV mass and MS (r = 0.21, p = 0.44).

#### HCM-HFpEF group

The mean MS for the HCM-HFpEF group was 12.68 ± 2.91 kPa. Only 2 patients had an MS value <8 kPa (6.46 kPa and 7.97 kPa). The correlation between age and MS values for this pathological group was low (r^2^ = 0.14, r = 0.37, p < 0.01). We found no difference between MYH7 and MYBPC3 mutation subgroups (p = 0.34).

#### Comparison between MS healthy and MS HCM-HFpEF groups

**Figure 4:**
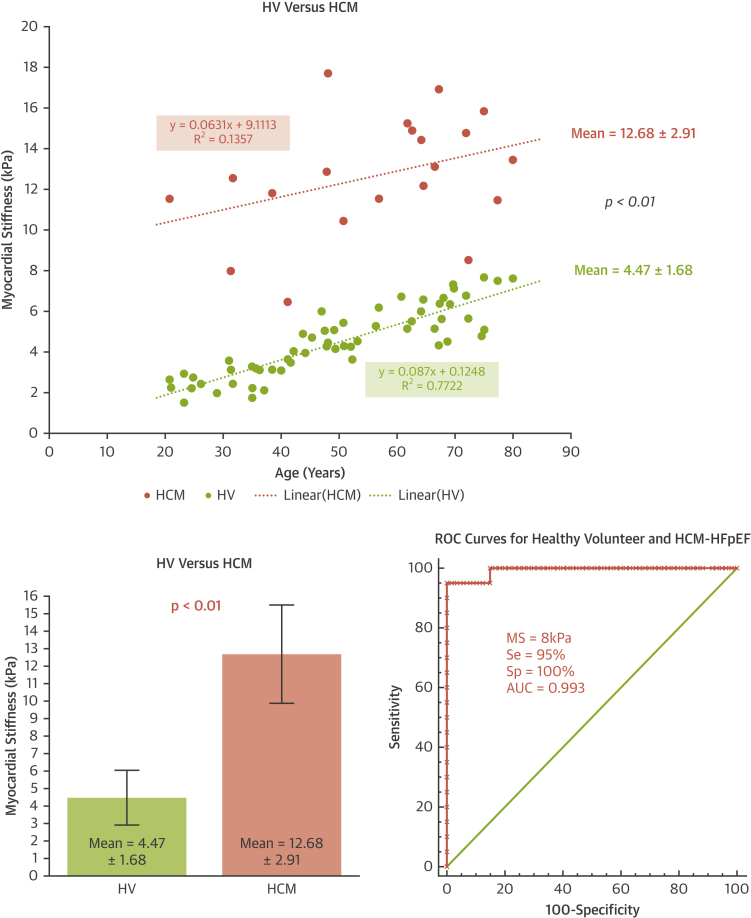
Myocardial Stiffness in HCM and HV Groups Comparison of Myocardial Stiffness between healthy volunteer group (HV) and hypertrophic cardiomyopathy with HFpEF group (HCM group). AUC = area under the curve; MS = myocardial stiffness; ROC = receiver-operating curve; Se = sensitivity; Sp = specificity; other abbreviations as in Figures 1 and 2.

There was a significant statistical difference between the MS healthy group and the MS HCM-HFpEF group (p < 0.01) (Figure 4). Based on the ROC curve analysis, the optimal cut-off value of MS for detection of HCM-HFpEF was 8 kPa (AUC = 0.993, sensitivity = 95%, specificity = 100%).

#### Correlation of MS with measures of diastolic function and other parameters

Positive correlations were found between MS and parameters in echocardiography (E/e′, r = 0.783, p < 0.01; E/Vp, r = 0.616, p < 0.01; left atrial volume index [LAVI], r = 0.623, p < 0.01) and CMR (LGE, r = 0.804, p < 0.01; myocardial T1 pre-contrast, r = 0.711, p < 0.01; myocardial T1 post-contrast, r = 0.595, p = 0.01; ECV fraction, r = 0.447, p = 0.03). Correlation of MS with other parameters is summarized in Table 2. There was no correlation between MS and the global strain (r = 0.37, p = 0.27) or the septal strain (r = 0.40, p = 0.09). There was no correlation between MS and BNP (r = 0.41, p = 0.06).

**Table 2:** Myocardial Stiffness Correlations

	r	p Value
Patient		
Age, yrs	0.881	<0.01
Sex, M/F	NA	NA
BMI, kg/m^2^	0.089	0.98
Systolic BP, mm Hg	0.207	0.33
Diastolic BP, mm Hg	0.154	0.63
NYHA functional class	0.677	0.02
Biology (blood explorations)		
CRP, mg/l	0.217	0.36
NTproBNP, pg/ml	0.413	0.06
Hematocrit, %	0.299	0.15
Echocardiography parameters		
LA surface, 4 cavities view, cm^2^	0.378	0.21
LAVI, ml/m^2^	0.623	<0.01
LVEF, %	0.204	0.45
LVEDD, mm	0.266	0.51
LVESD, mm	0.178	0.76
LV mass/BSA, g/m^2^	0.329	0.23
LV GS, %	0.378	0.27
ASB segment GS, %	0.420	0.09
ASB segment end diastolic thickness, mm	0.277	0.31
Peak E-wave, cm/s	0.304	0.30
E/A	0.506	0.01
e’ septal, cm/s	0.365	0.39
E/e’	0.783	<0.01
e’/a’ septal	0.452	0.07
E-wave DT, ms	0.511	0.02
IVRT, ms	0.361	0.14
Vp, cm/s	0.219	0.55
E/Vp	0.616	<0.01
PV S/D ratio	0.422	0.10
Cardiac magnetic resonance		
ASB segment end diastolic thickness, mm	0.325	0.17
LV mass/LVED volume, g/ml	0.388	0.11
Myocardial T1 pre-contrast, ms	0.711	<0.01
Blood T1 pre-contrast, ms	0.195	0.67
Myocardial T1 post-contrast, ms	0.595	0.01
Blood T1 post-contrast, ms	0.101	0.77
Focal LGE present, ASB segment	0.804	<0.01
Extracellular volume fraction, %	0.447	0.03

NA = not available; other abbreviations as in Table 1.

#### Reproducibility

Among the 15 HV patients who were re-evaluated 3 months later, there was no statistical difference with the initial assessment (mean MS = 4.26 ± 1.36 kPa; p = 0.67). Moreover, Bland-Altman analysis showed good agreement between measurements: MS +0.08 kPa (upper limit of agreement: +0.89 kPa; lower limit of agreement: −0.73 kPA) (Supplemental Figure 2).

### Fractional anisotropy

**Figure 5:**
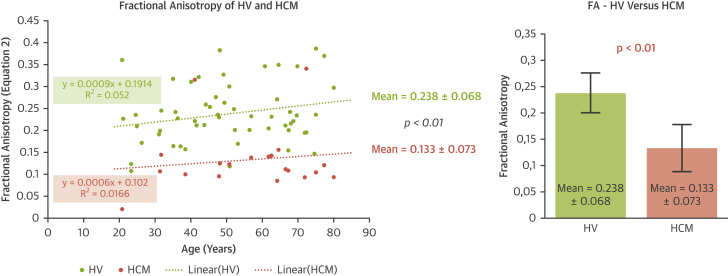
Fractional Anisotropy Comparison of fractional anisotropy (FA) between healthy volunteer group (HV) and hypertrophic cardiomyopathy with HFpEF group (HCM group).

The mean FA for the HV group (0.238 ± 0.068) was higher than that for the HCM-HFpEF group (0.133 ± 0.073; p < 0.01). Eighteen of 20 patients (90%) from the HCM-HFpEF group had an FA <0.155 whereas 56 of 60 patients (93%) from the HV group had an FA larger than this cut-off (AUC = 0.891, sensibility = 90%, specificity = 91.2%) (Figure 5).

## Discussion

In this study, MS was assessed quantitatively using noninvasive SWI in HV and HCM patients with HFpEF. To our knowledge, this is the first study to assess MS quantitatively and noninvasively in both HV and pathological cases (HCM-HFpEF). This study showed that SWI allows us to establish values of MS in a HV population, MS increases strongly with age in the normal heart, and there is a large difference in MS between HV and HCM-HFpEF groups (cutoff = 8 kPa).

In this study we were able to quantify MS aging. In the NORRE (Normal Reference Ranges for Echocardiography) study, Caballero et al. (26) also found a gradual change with age of the main echocardiographic parameters of the diastolic function. In this study, which analyzed 449 HV echocardiographs, the E/e′ ratio increased from an average of 6.9 ± 1.6 in 20- to 39-year-old subjects to an average of 9.7 ± 2.8 in 60- to 79-year-old subjects, a change of approximately 50% with a fairly linear evolution. Myocardial aging was also evaluated by CMR on a human population (27) or by invasive estimation on animal study (28). In 1991, Weger et al. (29) have well demonstrated that the age-induced physiological myocardial fibrosis impacts on the cardiac function, including the ability of the ventricle to relax during the diastolic filling (auxotonic relaxation). Regarding HV patients who participated in our study, we also found a linear evolution of MS, allowing us to establish the change of MS with age.

We also showed an MS difference between HV and HCM-HFpEF noninvasively. Zile et al. (30) have shown on myocardial histologic explorations of HFpEF patients that an increase in passive MS is due to an architectural modification (increase of collagen and titin). Moreover, in a systematic review on HCM published in 2002, Barry J. Maron (3) noted that the “LV myocardial architecture is disorganized […] with multiples intercellular connections often arranged on chaotic alignment and with expanded interstitial (matrix) collagen,” which is supported by previous work that tried to link myocardial histological explorations and MS (31). Our study shows that the abnormal MS of this characteristic pathological group (HCM-HFpEF) can be quantified noninvasively. In addition, SWI provided information on myocardial architecture through the analysis of the FA which revealed differences between the tissue architecture of the 2 groups, with a decrease of the physiological anisotropy in the HCM-HFpEF group. However, the clinical interest of this parameter must be further investigated and analyzed on more patients and other pathological groups.

Beyond the analysis of viscoelastic properties and of tissue structure, the comparison of MS with the recommended ultrasound parameters used to assess the diastolic function seems to show that this quantitative parameter could help to distinguish patients with diastolic dysfunction from others. Obviously, the diastolic function analysis remains complex and it would be unrealistic to think that 1 quantitative parameter could define the LV diastolic function. For example, LAVI >34 ml/m^2^ is a key structural alteration allowing the definition the diagnostic of HFpEF (19), but Caballero et al. (26) have also clarified that 15.1% of heathy people have a LAVI >34 ml/m^2^ whereas only 0.5% have an E/e′ ratio >15. Nevertheless, the MS assessment of patients with HFpEF has clearly helped to improve understanding of this disease (32). Performing the assessment noninvasively would refine our diagnostic capabilities and may help us to understand disease “at the bedside,” with a noninvasive approach.

Beyond the diagnostic contribution that the evaluation of MS by SWI could represent, the prospects of therapeutic follow-up could be interesting. The current finding is that there is no specific medical treatment of diastolic dysfunction in HCM. This is probably due to the fact that there is as yet no medical treatment with a high expected efficacy in HFpEF, as recalled in the recent European guidelines: “No treatment has yet been shown, convincingly, to reduce morbidity or mortality in patients with HFpEF” (19). Noninvasive MS assessment could be a major tool for the development of novel treatments of HCM and/or HFpEF. Thanks to this noninvasive MS marker, the impact of certain treatments (angiotensin-converting enzyme inhibitors or mineralocorticoid/aldosterone receptor antagonists, for example) could be evaluated quantitatively.

Finally, we have observed a good reproducibility of the MS assessment. Despite the small size of this analysis group (n = 15), which limits its interpretation, these results indicate that this technique could be used to evaluate a patient through longitudinal follow up. We did not re-evaluate the reproducibility of this technique on HCM-HFpEF groups because it is still difficult to estimate the impact of the disease evolution on the MS results, and the treatments of these patients were modified after the initial evaluation and could change their diastolic function (and maybe their myocardial structure).

### Study limitations

This was a monocentric study, and on a small sample. Therefore, it is difficult to extrapolate these results to a general population, which would require larger population groups. The gold standard of MS assessment remains an invasive measurement with a conductance catheter. From a regulatory point-of-view, this exploration could not be achieved on HV patients recruited exclusively for this study, notably because of the risks inherent to this invasive exploration. However, previous work on an animal model had shown a strong correlation between MS estimated by SWI and MS estimated through the end-diastolic strain-stress relationship 14, 15. In addition, the noninvasive echocardiographic evaluation of MS has been shown possible by extrapolating the LV volume-pressure curve (end-diastolic pressure volume relationship) knowing a single-beat point, based on Doppler and bidimensional data (volume) 33, 34, 35. It will be interesting in future studies to compare the MS estimated by SWI with this noninvasive method. Concerning the HCM-HFpEF group, no catheterization was provided in the management of these patients during the time of the study. Another limitation of our study is the local evaluation of MS by the SWI. Indeed, we compared global functional parameter (E/e′, E/Vp, or LAVI) with a segmental parameter (MS of the ASB segment). Nonetheless, the same segment was analyzed for all people included in the study. This study was limited to the basal septum. The main reason is that we used a conventional phased array with an elevation focus at 70 mm which limited the generation of the shear waves at higher depth. To address other segments, the development of dedicated probes will be required.

## Conclusions

In this study, we quantitatively assessed the end-diastolic MS in adult HV patients using SWI in the HV and sarcomeric HCM with HFpEF patients. MS was found to increase with age, and a cut-off of 8 kPa allowed clear differentiation of these 2 groups. The FA obtained by SWI reflected the underlying tissue structure modifications. Thanks to this ultrasound technology, the noninvasive assessment of MS enables a new diagnostic option in cardiology. Future studies will aim to evaluate MS on other heart diseases, such as isolated HFpEF, HF with reduced EF, hypertension, diabetes cases with HFpEF, or other cardiomyopathies to determine the impact of this parameter in clinical practice.

Perspectives **COMPETENCY IN MEDICAL KNOWLEDGE:** This study is the first, to our knowledge, to quantify MS in patients using noninvasive SWI. It opens the path to the clinical evaluation of LV function using a new parameter relatively independent of loading. We expect this parameter to be robust, stable, and representative of the myocardial LV diastolic function.

**TRANSLATIONAL OUTLOOK:** Future studies should address the evaluation of this new parameter in systolic and diastolic heart failure patients and whether this parameter could improve the diagnosis and prognosis of this population.
