# Supplementary material for: Myocardial Stiffness Evaluation Using Noninvasive Shear Wave Imaging in Healthy and Hypertrophic Cardiomyopathic Adults
Source: JACC Cardiovasc Imaging. 2019 Jul;12(7):1135–45. doi: 10.1016/j.jcmg.2018.02.002 (PMC6603249; doi:10.1016/j.jcmg.2018.02.002)
Supplement: Supplemental Data [file mmc1.docx]

**SUPPLEMENTAL METHODS**

*Myocardial stiffness evaluation using shear wave imaging*

A short push (300 µs) of focused ultrasound was transmitted by a diagnostic ultrasonographic probe (phased array, 2.75-MHz central frequency; SuperSonic Imagine, Aix-en-Provence, France) to induce micrometric tissue displacements in a small zone of the myocardium by acoustic radiation force. The push was generated in the left ventricular endocardium. In response to that transient mechanical excitation, a shear wave was generated in the low–kHz-frequency range and propagated in the myocardium at velocities from 1 to 10 m/s, depending upon the intrinsic tissue stiffness. Tissue velocity maps were computed offline for each frame by using in-phase quadrature frame to frame cross-correlation^1^. Myocardial wall motion was removed by subtraction of the average wall motion during the acquisition, bringing to light tissue motion induced solely by the shear wave. Shear wave velocity was computed at the depth of the mid-wall myocardium using spatiotemporal data from shear wave propagation. Finally, the shear modulus µ (i.e., stiffness) was derived using the equation:

µ = ρc^2^ (kPa)

where c is the shear wave velocity, and ρ is the volume mass of the tissue.

*Fractional Anistropy*

FA was defined using two shear wave speed (SWS) measurements performed in orthogonal propagation directions (long axis and short axis views) using the formula published by Lee et al^1^ in 2011:

FASWI = $\sqrt{2}$ $\frac{\sqrt{\left( SWSsa-SWSm \right)^{2}+(SWSla-SWSm)^{2}}}{\sqrt{SWSsa^{2}+SWSla^{2}}}$ (Equation 1)

where SWSsa and SWSla were respectively the shear wave speed in short axis and in long axis, and SWSm was the mean shear wave speed.

*References*

1. Bercoff J, Tanter M, Fink M. Supersonic shear imaging: a new technique for soft tissue elasticity mapping. *IEEE Trans Ultrason Ferroelectr Freq Control*. 2004;51(4):396-409. doi:10.1109/TUFFC.2004.1295425.

2. Lee W-N, Larrat B, Pernot M, Tanter M. Ultrasound elastic tensor imaging: comparison with MR diffusion tensor imaging in the myocardium. *Phys Med Biol*. 2012;57(16):5075-5095. doi:10.1088/0031-9155/57/16/5075.

**Supplemental Figures**

**Figure 1: Correlation between echocardiographic parameters (E/A, E/e’, E/Vp) and age on heathy volunteers**


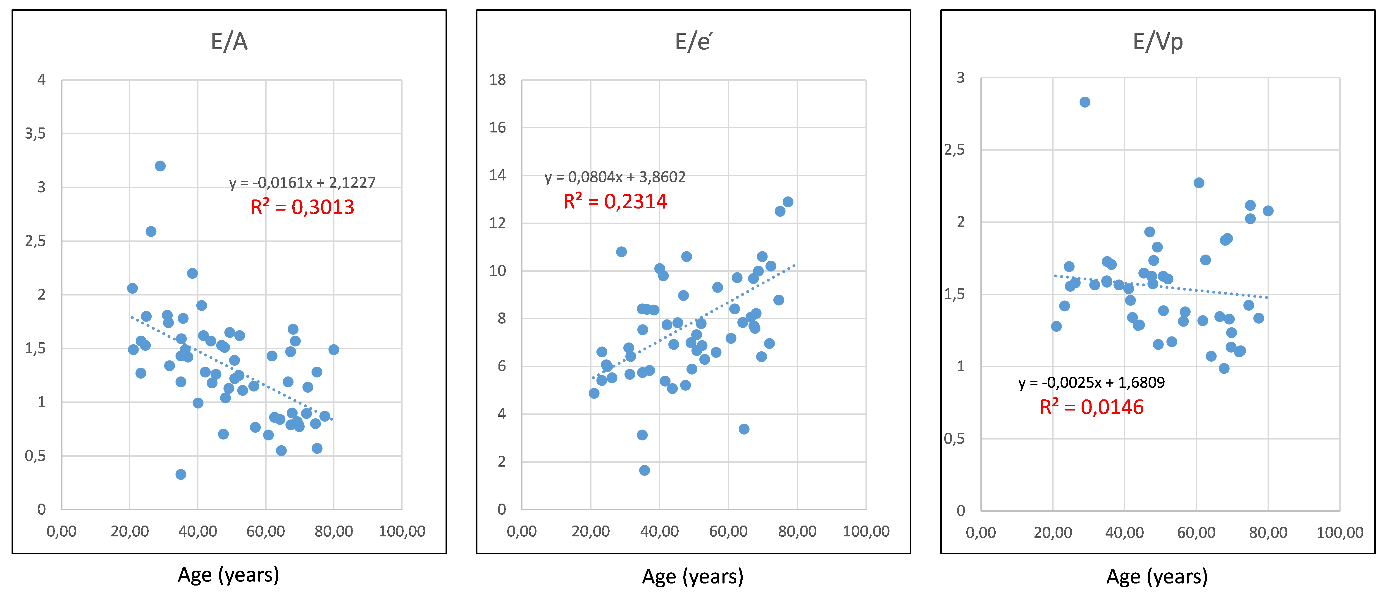


**Figure 2: Bland-Altman-Plots for myocardial stiffness analysis**


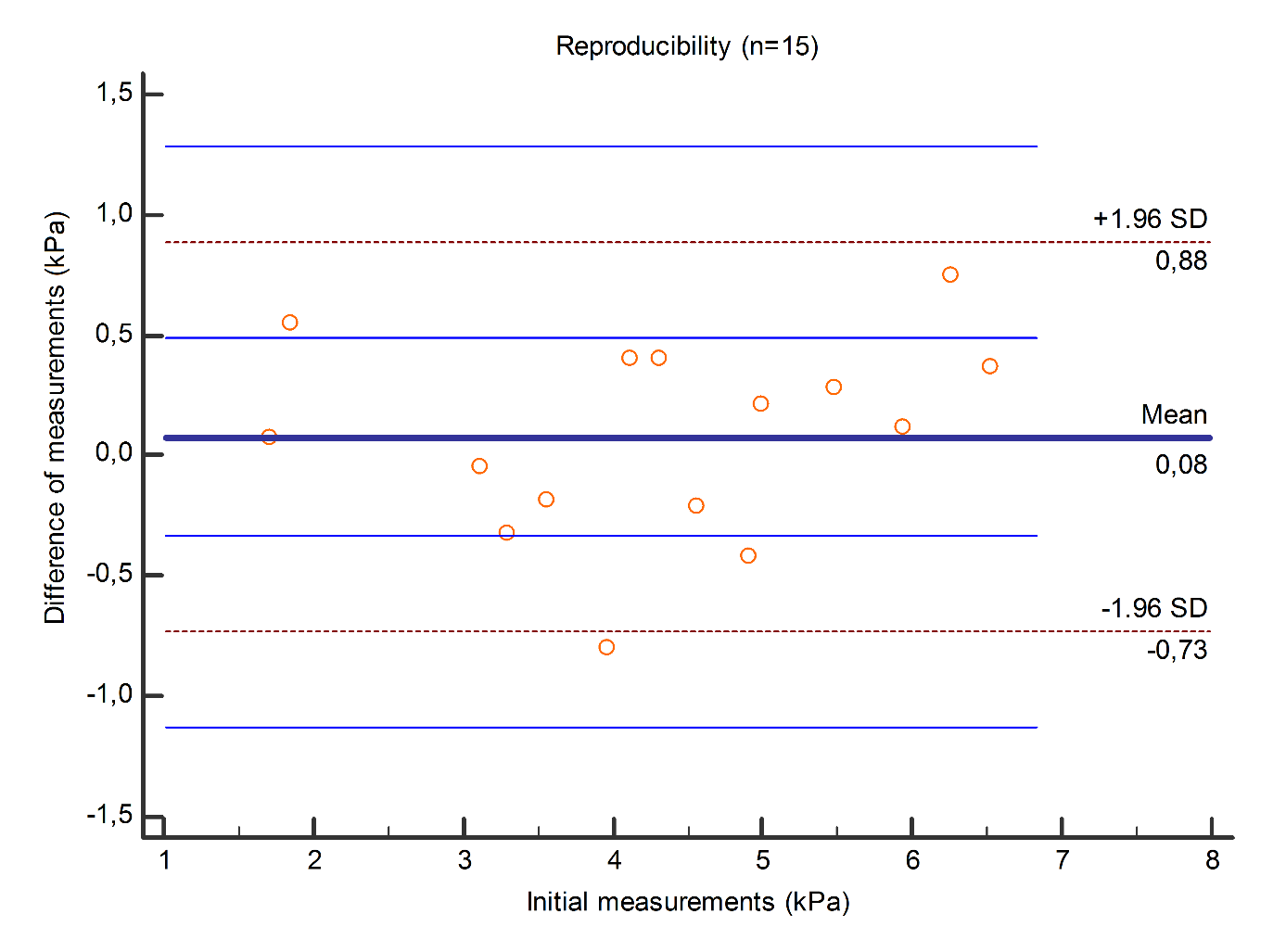


Bland-Altman-Plots with mean difference of measurements (three months later) and limits of agreement for the HV sub-group (n=15). The mean difference is equal to +0.08 kPa (upper limit of agreement [ULA]: +0.89 kPa; lower limit of agreement [LLA]: -0.73 kPA).
